# Targeting opportunities presented by the pyrimidine biosynthesis pathway in *Mycobacterium tuberculosis:* a brief review

**DOI:** 10.1042/BST20253113

**Published:** 2026-01-12

**Authors:** Marta Alberti, Riccardo Miggiano

**Affiliations:** 1 Department of Pharmaceutical Sciences, Via G. Bovio 6, University of Piemonte Orientale, Novara, 28100, Italy

**Keywords:** drug design, drug discovery, *Mycobacterium tuberculosis*, pyrimidine biosynthesis pathway, tuberculosis therapies

## Abstract

*Mycobacterium tuberculosis* (MTB) is the etiologic agent of tuberculosis (TB) in humans, an infectious disease that continues to be a significant global health concern. The long-term use of multiple anti-tubercular agents may result in patient non-compliance and increased drug toxicity, which could contribute to the emergence of drug-resistant MTB strains that are not susceptible even to second-line available drugs. It is therefore imperative that new antitubercular drugs and vaccines are developed. The peculiar traits of MTB, such as the biochemical and structural features of vital metabolic pathways, can be assessed to identify possible targets for drug development. Enzymes involved in pyrimidine metabolism may be suitable drug targets for TB, given that this pathway is essential for mycobacteria and comprises enzymes that differ from those found in humans. Here, we focused on reviewing the state of the art concerning the therapeutic opportunities presented by the pyrimidine biosynthetic pathway (PBP) as a potential source of enzymes that could be targeted for the treatment of TB. We selected essential enzymes belonging to the PBP for which we identified the existence of a drug discovery pipeline at both the preclinical and clinical levels. Moreover, we emphasize the biochemical and structural characteristics that are pertinent to the development of pharmaceutical agents. These include the molecular details that can ensure selectivity towards the pathogen’s proteins.

## Introduction

*Mycobacterium tuberculosis* (MTB) is the leading causative agent of tuberculosis (TB) and, until the Coronavirus Disease 19 (COVID-19) pandemic, was the world’s primary infectious killer [1]. According to the last Global Tuberculosis Report, about 1.25 million deaths in 2023 were caused by TB, and the COVID-19 emergency further complicated the disease management [1]. The rising number of drug-resistant strains, delays in case notifications, and the poverty of most affected countries continue to hinder TB control and eradication. Current treatments rely on long multidrug regimens, lasting four to nine months, using first-line agents that target MTB’s unique cell wall, including rifampicin, isoniazid, pyrazinamide, streptomycin, and ethambutol [2]. However, rapid microbial mutation contributes to the emergence of drug-resistant strains, highlighting the need for new therapeutic targets. Accordingly, investigating and targeting metabolic pathways essential for MTB provide promising alternatives for drug development. With this aim, the present review focuses on key components involved in the pyrimidine biosynthetic pathway (PBP), which are critical building blocks for DNA/RNA synthesis. Here, we focused on molecular targets thought to represent new therapeutic opportunities; specifically, we delved into crucial pathways for MTB viability, shedding light on enzymes whose druggability has been explored in drug discovery studies.

## Pyrimidine biosynthesis in MTB

Pyrimidines are essential components of many biomolecules, making their biosynthetic pathways pivotal to cellular metabolism [3]. Targeting these metabolic cascades can be approached either by inhibiting key enzymes or transporters, resulting in lethal pyrimidine depletion, or by using pyrimidine analogs that, once integrated into nucleic acid structures, exert toxicity through enzyme inhibition or *via* their metabolites [4]. Pyrimidines are synthesized through two primary pathways: the *salvage* and the *de novo* biosynthesis, and the relative contribution of these sources depends on cell type and metabolic cell state [5]. In particular, while the *salvage* PBP minimizes energetic costs for cells, *de novo* PBP is more resource-intensive [6]. MTB possesses the complete *apparatus* for both the *salvage* and *de novo* synthesis, as identified through the complete sequencing of the MTB genome [7]. Biochemical insights into these processes are described below.

### Pyrimidine salvage pathway

**Figure 1:**
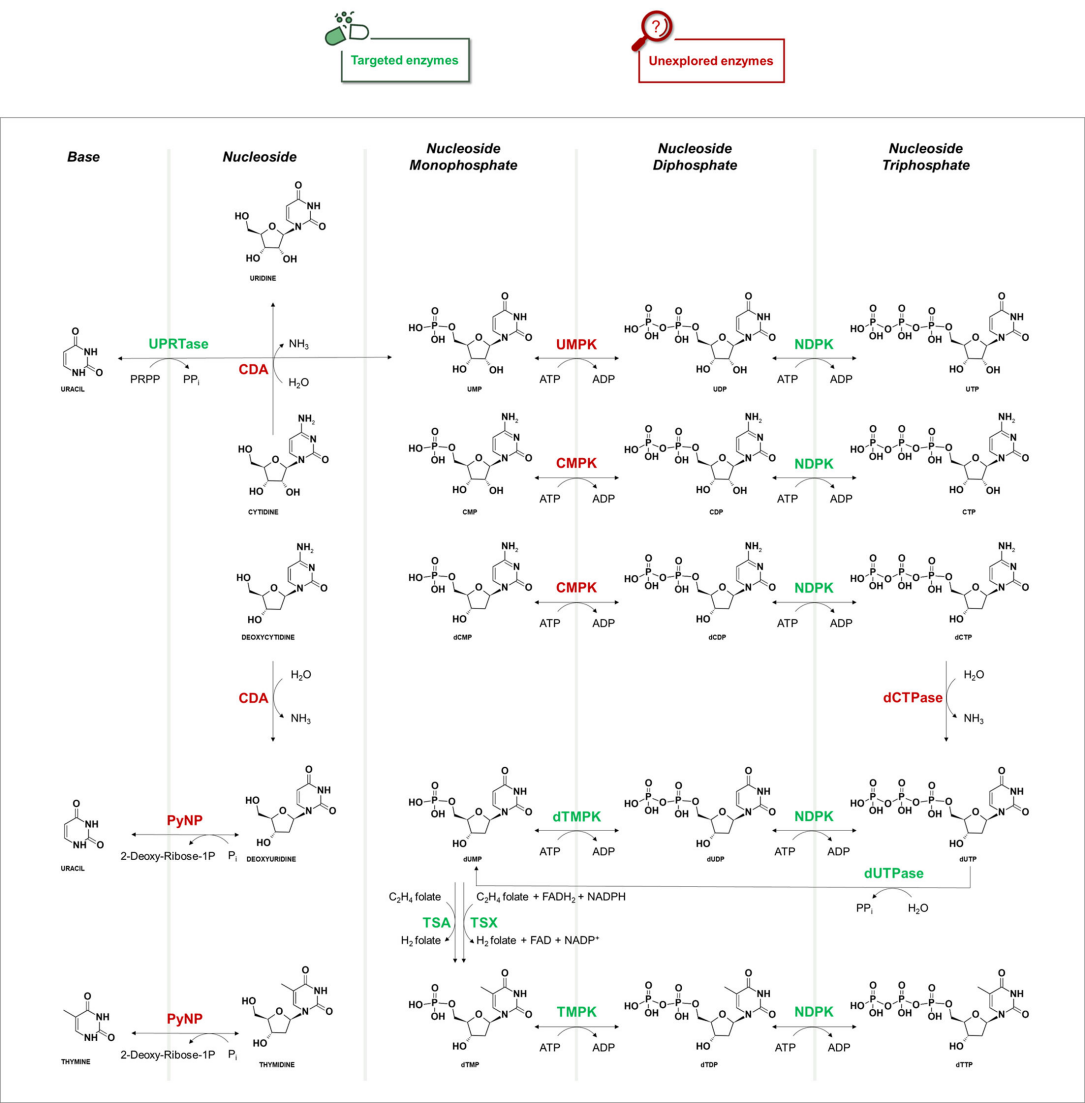
Pyrimidine *salvage* pathway in *Mycobacterium tuberculosi*s with representation of all the enzymes involved. The diagram illustrates the metabolic route through which pyrimidine bases and nucleosides are salvaged and converted into nucleotides – mono-, di-, and triphosphates. Green-highlighted components represent enzymes that have already been investigated as potential targets in TB drug discovery. In contrast, red-highlighted components indicate enzymes that have not yet been the focus of inhibition studies or are considered non-essential based on available functional or genetic data. Enzymes and intermediates involved in this pathway are labeled accordingly. Abbreviations: ADP, adenosine diphosphate; ATP, adenosine triphosphate; C2H4folate, 5,10-methylenetetrahydrofolate; CDA, cytidine deaminase; CDP, cytidine diphosphate; CMP, cytidine monophosphate; CMPK, cytidine monophosphate kinase; CTP, cytidine triphosphate; dCDP, deoxycytidine diphosphate; dCMP, deoxycytidine monophosphate; dCTP, deoxycytidine triphosphate; dCTPase, deoxycytidine triphosphate deaminase; dUTPase, deoxyuridine triphosphatase; dTMP, deoxythymidine monophosphate; dTMPK, deoxythymidine monophosphate kinase; dTDP, deoxythymidine diphosphate; dTTP, deoxythymidine triphosphate; dUDP, deoxyuridine diphosphate; dUMP, deoxyuridine monophosphate; dUTP, deoxyuridine triphosphate; FAD, flavin adenine dinucleotide; FADH2, reduced flavin adenine dinucleotide; H2folate, dihydrofolate; NADP+, nicotinamide adenine dinucleotide phosphate; NADPH, reduced nicotinamide adenine dinucleotide phosphate; NDPK, nucleoside diphosphate kinase; PPi, inorganic pyrophosphate; PRPP, 5-phospho-alpha-D-ribose 1-diphosphate; PyNP, pyrimidine nucleoside phosphorylase; TSA, thymidylate synthase *thyA*; TSX, thymidylate synthase *thyX*; UDP, uridine diphosphate; UMP, uridine monophosphate; UMPK, uridine monophosphate kinase; UPRTase, uracil phosphoribosyltransferase; UTP, uridine triphosphate.

The *salvage* PBP recycles pyrimidine bases and nucleotides from degraded DNA/RNA. As illustrated in Figure 1, several enzymes contribute to nucleotide turnover [8], some of which present therapeutic opportunities for cancer [9] and infectious diseases [10]. Focusing on antitubercular application, nutrient starvation is critical for the long-term survival of mycobacteria during latent infection, particularly within granulomas, where oxygen and nutrient availability are limited [11–13]. In this context, pyrimidine reutilization becomes essential. Below, we outline enzymes involved in the *salvage* PBP of MTB, focusing on those essential for mycobacterial survival whose druggability potential has been explored in inhibition studies (Table 1).

**Table 1:** List of gene names, corresponding protein abbreviations, MTB-reported loci, E.C. codes, UniProt codes, and the relevance of each gene for MTB survival. The final column indicates whether inhibition studies have been conducted (YES) or not (NO) for each respective enzyme. As mentioned in the manuscript, non-essential genes (/) were not described as potential drug targets. *Data derived from the *Mycobrowser* database

Gene name	Protein encoded	MTB locus	E.C. code	UniProt code	Relevance*	Druggability
*cdd*	CDA	Rv3315c	3.5.4.5	P9WPH3	Non-essential gene [14]	/
*cmk*	CMPK	Rv1712	2.7.4.14	P9WPA9	Non-essential gene [14]	/
*dcd*	dCTP*ase*	Rv0321	3.5.4.13	P9W917	Non-essential gene [14]	/
*dut*	dUTP*ase*	Rv2697c	3.6.1.23	P9WNS5	Essential gene [14]	YES [15–24]
*tmk*	dTMPK	Rv3247c	2.7.4.9	P9WKE1	Essential gene [14]	YES [25–49]
*ndkA*	NDPK	Rv2445c	2.7.4.6	P9WJH7	Essential gene [14]	YES [50–58]
*deoA*	PyNP	Rv3314c	2.4.2.4	P9WFS1	Non-essential gene [14]	/
*pyrH*	UMPK	Rv2883c	2.7.4.22	P9WHK5	Essential gene [14]	NO
*upp*	UPRT*ase*	Rv3309c	2.4.2.9	P9WFF3	Non-essential gene [14]	/
*thyA*	TSA	Rv2764c	2.1.1.45	P9WFR9	Essential gene [14]	YES [59–68]
*thyX*	TSX	Rv2754c	2.1.1.148	P9WG57	Essential gene [14]	YES [14,69–75]

#### Deoxyuridine triphosphatase (dUTPase)

**Figure 2:**
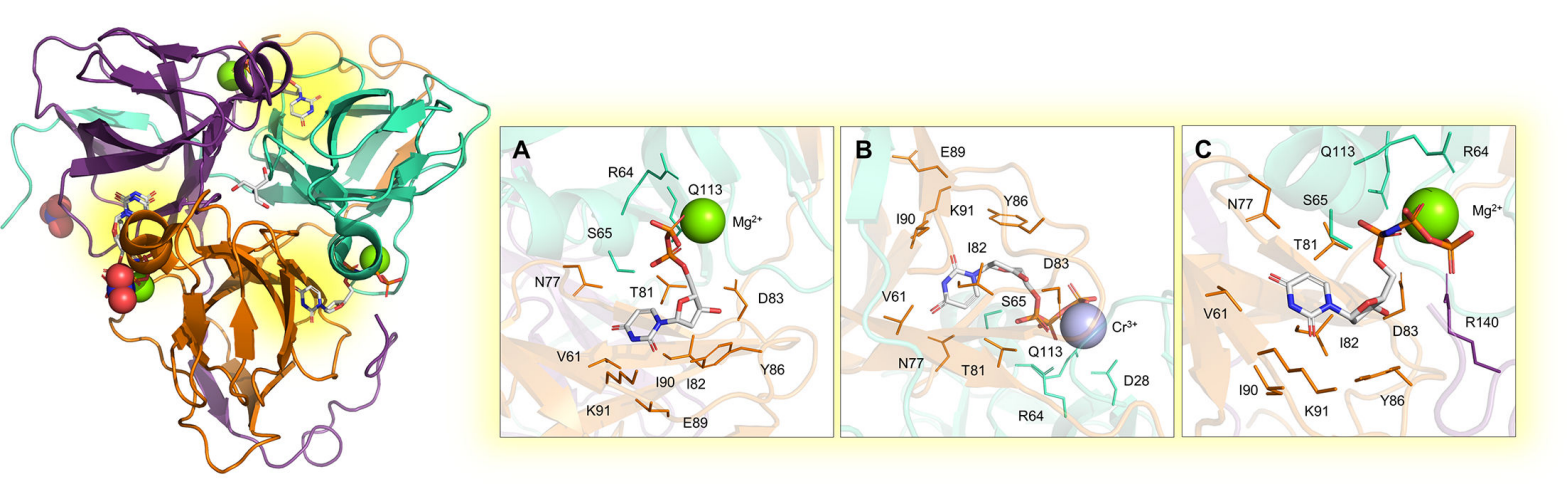
Experimental structure of the homotrimeric MTB dUTP*ase* enzyme. The structures were solved by X-ray crystallography in the apo form and in complex with: (**A**) Mg²^+^ and dUDP (PDB: 1SLH [16]; (**B**) Cr³^+^ and dUTP (PDB: 1SM8 [16]); (**C**) Mg²^+^ and 2′-Deoxyuridine 5′-α,β-imido-triphosphate (PDB: 1SJN [16]. Active sites are highlighted as yellow spots at the interface of each monomer.

The dUTP*ase* protein hydrolyzes dUTP into dUMP, releasing PP_i_ and employing Mg^2+^ as a cofactor. This enzyme plays a pivotal role in regulating cellular levels of dUMP, the obligatory precursor for thymine-based nucleotides. Since most DNA polymerases are unable to distinguish between uracil and thymine, an elevated dUTP/dTTP ratio can cause toxicity through uracil incorporation into DNA [76]. A deficiency of dTTP, with subsequent incorporation of dUMP into DNA, activates DNA-repair mechanisms aimed at replacing uracil with thymine. However, overactivation of the uracil-excision repair pathway can ultimately lead to double-stranded DNA breaks, a phenomenon known as thymine-less cell death [15]. Given that the genes encoding for dCMP deaminase (which converts dCMP into dUMP) and thymidine kinase (which converts thymidine to dTMP) have not been identified in the MTB genome, dUTP*ase* emerges as the sole enzyme producing thymidine-based nucleotides. Therefore, dUTP*ase* represents a promising target for TB treatment, as thymidine-nucleotide synthesis relies exclusively on its activity. The crystal structure of the mycobacterial wild-type protein was obtained both free and ligand-bound (Figure 2A and B), including with a non-hydrolyzable substrate analog [16–18] (Figure 2C). Structural data indicate that the homotrimeric structure is crucial for dUTP*ase* activity, and mutations that alter trimer conformation disrupt active-site interactions, impacting catalytic efficiency [18–23]. Given its essential biological role, MTB dUTP*ase* has been proposed as a promising target for TB treatment [23,24]. Despite the structural similarity with the human dUTP*ase* posing significant challenges in the design of selective inhibitors, a species-specific peculiarity in the trimer interface channel related to S78-P79 insertion can be exploited for selective drug design. Indeed, this insertion results in a constriction of the diameter of the trimer interface channel, reducing the volume from 673 Å^3^ in the human protein to 309 Å^3^ in the MTB enzyme. Furthermore, the side chains contouring the channel exhibit notable differences between species, and the presence of a Tris buffer molecule can drive the design of inhibitors for the mycobacterial protein [16].

#### Thymidylate synthase thyA (TSA)

**Figure 3:**
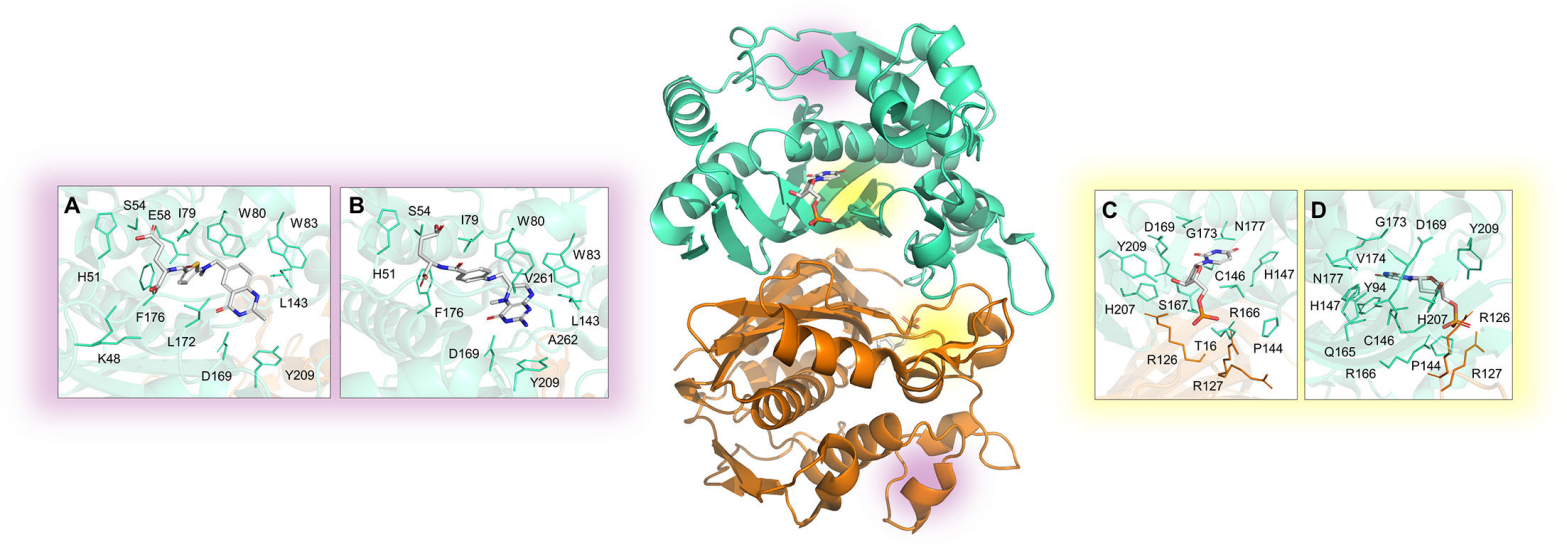
Experimental structure of the homodimeric MTB TSA enzyme. The structures were solved by X-ray crystallography in the apo form and in complex with: (**A**) Raltitrexed (PDB: 4FOX); (**B**) Pemetrexed (PDB: 4FQS) located in the C_2_H_4_ folate cofactor-binding site (highlighted in violet in structure); (**C**) the substrate dUMP (PDB: 3QJ7); (**D**) the substrate analog FdUMP (PDB: 4FOA) located in the substrate binding site (highlighted in yellow in the structure).

Involved in *salvage* and *de novo* pathways, TSA catalyzes the reductive methylation of dUMP to dTMP, utilizing C_2_H_4_ folate as a methyl donor. Inhibition of TSA leads to dTMP depletion and dUMP accumulation, causing increased cell death due to the misincorporation of uracil into double-stranded DNA [77]. This cytotoxic effect is mitigated by the activity of thymidine kinase*,* which converts thymidine into dTMP, thereby increasing dTTP levels. However, the absence of thymidine kinase in the MTB genome underscores the critical role of TSA in mycobacterial survival [59]. Hence, TSA may be an interesting target for antiproliferative and antimicrobial therapies [60], and its inhibition has been investigated in anticancer treatments involving compounds such as 5-Fluorouracil [61], Pemetrexed [62], Raltitrexed [63], and Nolatrexed [64], with the latter two evaluated as potential inhibitors of the MTB enzyme (Figure 3A and B). These treatments likely act through both dUMP accumulation and dTMP depletion. However, the highly conserved sequence within the active site of human and bacterial TSA (50% sequence identity) complicates selective drug design. Studies indicate that phenolphthalein analogs can selectively inhibit *Lactobacillus casei* TSA by targeting unique active-site residues (E84, T85, E88, and res90-139), which are absent from the human enzyme [60]. Additionally, certain phthalein derivatives preferentially inhibit TS from *Pneumocystis carinii* or *Cryptococcus neoformans* [65]. In the context of TB disease, the functional and structural characteristics of MTB TSA enzyme in complex with the substrate have been defined (Figure 3C), while Hunter and co-workers [66] demonstrated that FdUMP (substrate analog) and cp1843U89 (C_2_H_4_folate analog) effectively inhibit the enzyme, with K_i_ of 2 nM and 18 nM, respectively. Moreover, the crystal structure of MTB TSA enzyme in complex with FdUMP is available in the Protein Data Bank (PDB) (Figure 3D). Nevertheless, these compounds also inhibit human TSA, precluding their use due to non-specificity concerns [66,67]. Despite this, targeting MTB TSA remains a promising area for future drug development by leveraging structural and functional differences between bacterial and human enzymes. In this sense, crystallographic studies of recombinant MTB TSA can drive future drug discovery.

#### Thymidylate synthase *thyX* (TSX)

**Figure 4:**
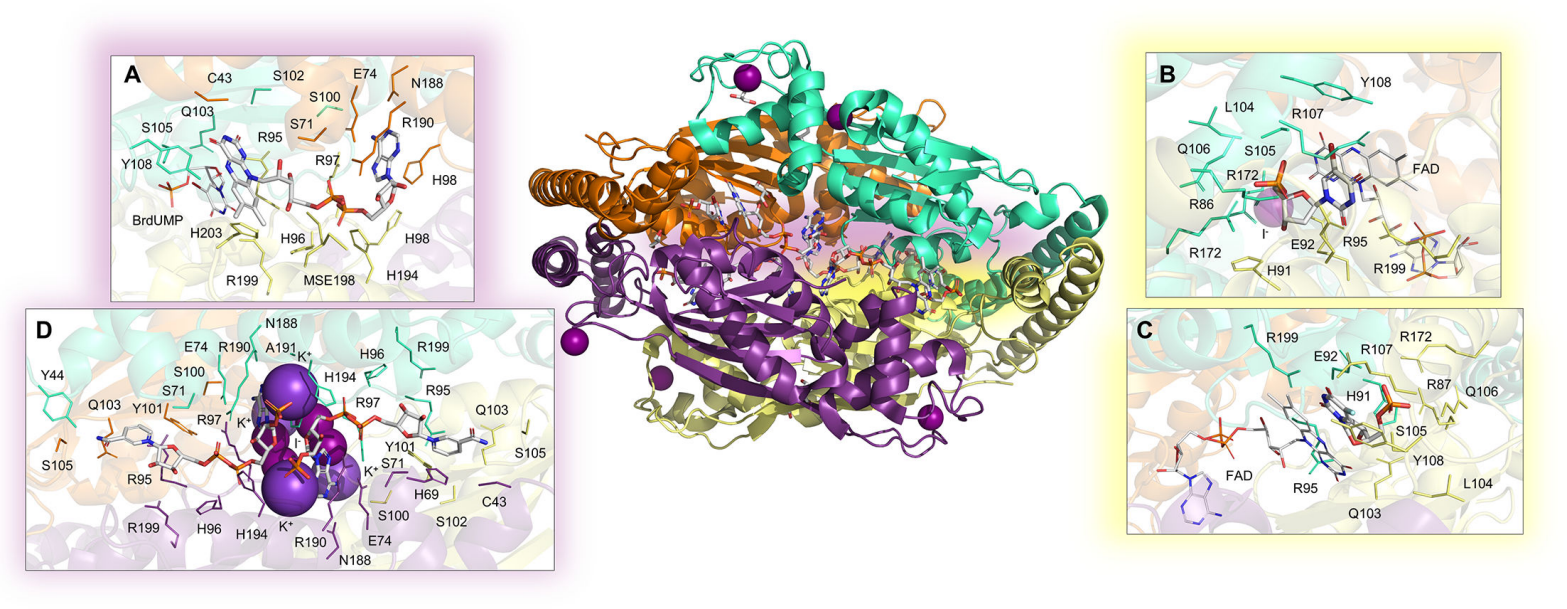
Experimental structure of the homotetrameric MTB TSX enzyme. The structures were solved by X-ray crystallography in complex with: (**A**) the cofactor FAD; (**B**) the substrate analog BrdUMP (PDB: 2AF6 [68]); (**C**) the substrate analog FdUMP (PDB: 3GWC [69]; (**D**) K^+^, I^−^ ions and NADP (PDB: 2GQ2 [70]), with NADP occupying the cofactor-binding site. The substrate binding site is highlighted as a yellow spot in the structure, whereas the cofactor binding site is highlighted in violet.

MTB possesses two genes, *thyA* and *thyX*, encoding proteins involved in dTMP production. Unlike TSA, TSX is a flavin-dependent synthase, characterized by a typical ‘TSX-motif’ with no direct similarity to TSA. The mycobacterial enzyme has been widely characterized, and its structure has been solved in complex with the cofactor FAD [68] (Figure 4A), the substrate analogs BrdUMP [68] and FdUMP [69] (Figure 4B and C), and NADP+ [70] (Figure 4D). High-throughput screening studies and substrate-based inhibitor design have produced several promising molecules with good potency profiles [71–74]. The most successful scaffold was optimized through a structure-activity-relationship (SAR)-driven approach, resulting in the identification of a lead compound with an IC_50_ of 0.69 μ M. Starting from the dUMP structure, Herdewijn and collaborators explored structural variations at C-5 of the uracil moiety, developing a novel series of 5-alkynyl dUMP analogs, with the most potent showing an IC_50_ of 0.91 μ M against MTB TSX [71]. Furthermore, the group conducted a systematic SAR and docking studies based on experimentally solved structures (Figure 4), revealing 5-undecyloxymethyl-2′-deoxyuridine-5′-monophosphate with an IC_50_ of 8.32 μ M [73] against the mycobacterial protein. These studies confirm the pharmacological potential of the enzyme, as the *thyX* gene is absent from humans, thereby rendering it a promising target for selective inhibitor development. Although the *thyA* and *thyX* genes are both reported as essential for mycobacterial growth [14], their specific metabolic roles and the vulnerability of MTB upon their inhibition remain unclear. It is unknown whether blocking a single enzyme is sufficient to kill MTB during growth or latency, whether one gene can compensate for inhibition of the other, whether cross-resistance may arise between inhibitors targeting each enzyme, or whether dual inhibition is required to fully disrupt dTMP synthesis through a synthetic-lethality strategy [75].

#### Deoxythymidine monophosphate kinase (dTMPK)

**Figure 5:**
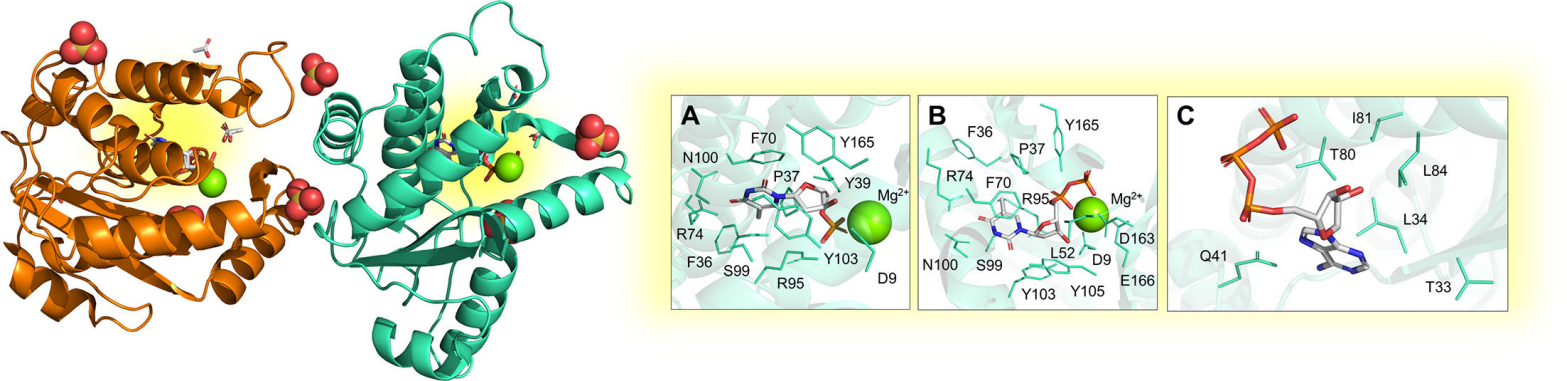
Experimental structure of the homodimeric MTB dTMPK enzyme. The structures were solved by X-ray crystallography in complex with: (**A**) Mg²^+^ and the substrate dTMP (PDB: 1G3U [28]); (**B**) Mg²^+^ and the product dTDP (PDB: 1GTV [29]; (**C**) Mg²^+^ and the cofactor ATP (PDB: 1N5I [30]. Substrate/cofactor-binding sites are highlighted in yellow in the structure.

While in eukaryotes the phosphorylation of nucleoside monophosphates is carried out by a single enzyme [78], in prokaryotes the process is catalyzed by distinct enzymes, namely UMPK and CMPK, specific for UMP and CMP, respectively [25]. Additionally, bacteria possess dTMPK, which phosphorylates both dTMP and dUMP (with lower affinity), using Mg^2+^ and ATP as a phosphate donor, to form dTDP and dUDP [26,27]. Situated at the junction of both *salvage* and *de novo* pathways, dTMPK is essential for DNA synthesis and represents an attractive target for the development of selective drugs against MTB, particularly given its low sequence identity with human dTMPK (23%). However, the highly conserved substrate-binding region complicates selective inhibitor design. The 3D structure of the homodimeric protein has been solved in complex with the substrate dTMP [28] (Figure 5A), the product dTDP [29] (Figure 5B), the ATP cofactor [30] (Figure 5C), and various nucleotide analogs [31–35], enabling the identification of promising scaffolds. Unlike *E. coli* or human dTMPKs, MTB dTMPK is competitively inhibited by 3′-azido-3′-deoxythymidine monophosphate, likely due to impaired Mg^2+^ binding observed in the co-crystal structure [36]. Extensive SAR studies have yielded two main classes of MTB dTMPK inhibitors: thymine-containing and non-thymine-containing molecules. Thymine-containing inhibitors are further classified into thymine nucleoside [27,28,35–38] and non-nucleoside [33,39–46] derivatives, the latter currently under evaluation in clinical trials as anti-TB agents [47,48] (see Table 2). Recent pharmacophore models identified flexible phthalimide and isoindoline moieties as advantageous for effective interactions within the enzyme’s active site [49]. Compared with previously reported inhibitors with μ M potency [79], these structural optimizations, along with additional substitutions, positively contributed to the interactions with key residues (K13, R14, R153, and Y39), achieving a remarkable potency of 0.15 nM for compound THMA34 [49]. Collectively, these findings underscore a promising direction for the development of new anti-TB agents capable of translating potent *in vitro* inhibition into effective *in vivo* activity.

**Table 2:** Chemical structures of co-crystallized compounds within the MTB dTMPK active site and corresponding PDB codes. The inhibitors are classified into two main categories: thymine-containing inhibitors (further divided into thymine nucleosides and non-nucleosides) and non-thymine-containing inhibitors

Thymine-containing inhibitors			
Compound	PDB code	Nucleoside derivatives	Non-nucleoside derivatives
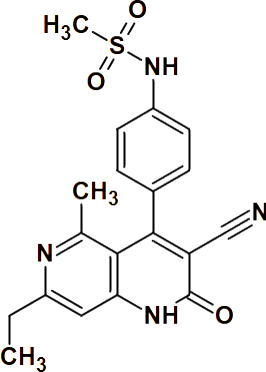	1 N5J [30]	YES	/
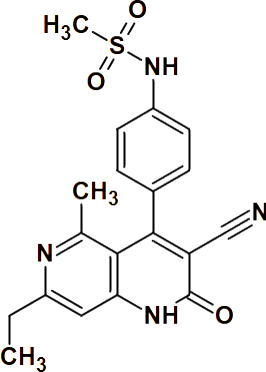	1MRN [31]	YES	/
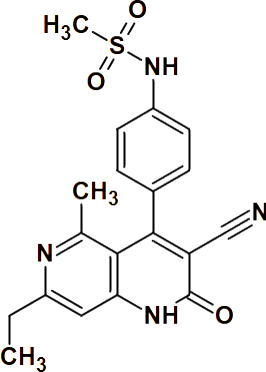	1MRS [31]	YES	/
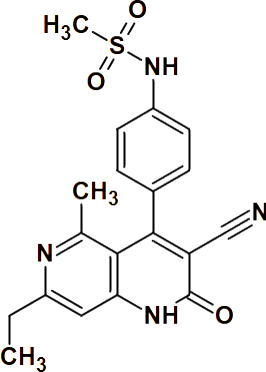	5NQ5 [33]	/	YES
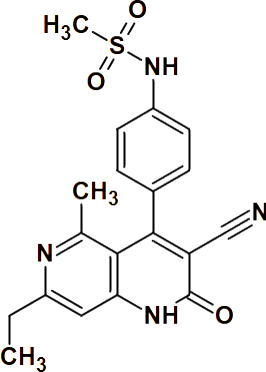	5NR7 [33]	/	YES
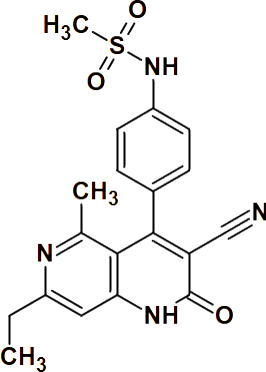	5NRN [33]	/	YES
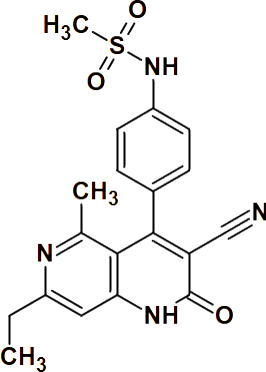	5NRQ [33]	/	YES
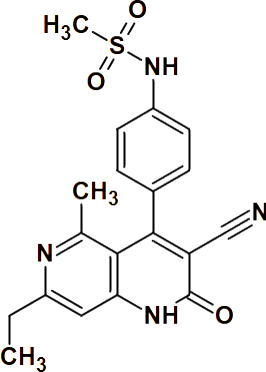	6YT1 [34]	/	YES
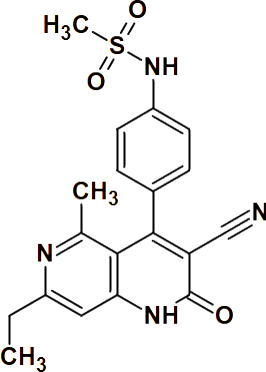	1 W2H [36]	YES	/
**Non-thymine-containing inhibitors**			
Compound	PDB code		
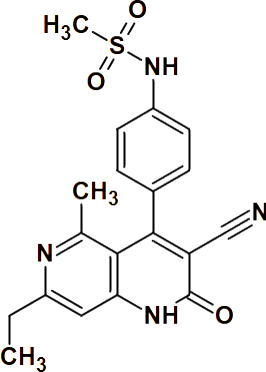	4UNN [32]		
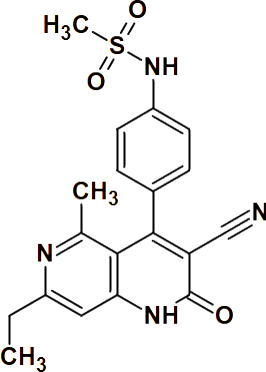	4UNP [32]		
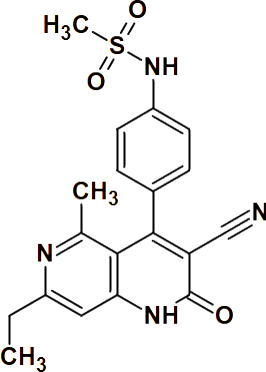	4UNQ [32]		
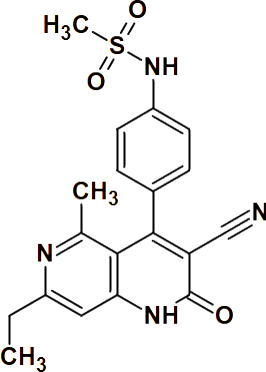	4UNR [32]		
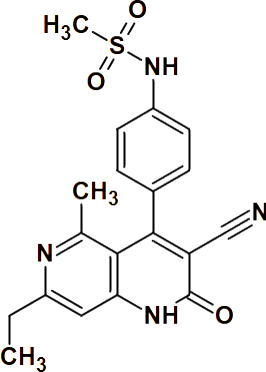	4UNS [32]		

#### Nucleoside diphosphate kinase (NDPK)

The NDPK enzyme has been fully characterized [50–56] and catalyzes the phosphorylation of nucleoside diphosphates ((d)NDPs) into triphosphates ((d)NTPs), utilizing ATP as the phosphoryl donor. Importantly, NDPK is essential for MTB survival within host macrophages, as it supports bacterial infection by helping the pathogen evade the host immune response [50,51]. As an ATP-utilizing enzyme, NDPK is secreted into the extracellular environment by MTB, where it regulates ATP-induced cell death in infected macrophages [57]. Hence, deletion of the *ndkA* gene has been considered in the design of a live attenuated vaccine against TB [58].

### Pyrimidine *de novo* pathway

**Figure 6:**
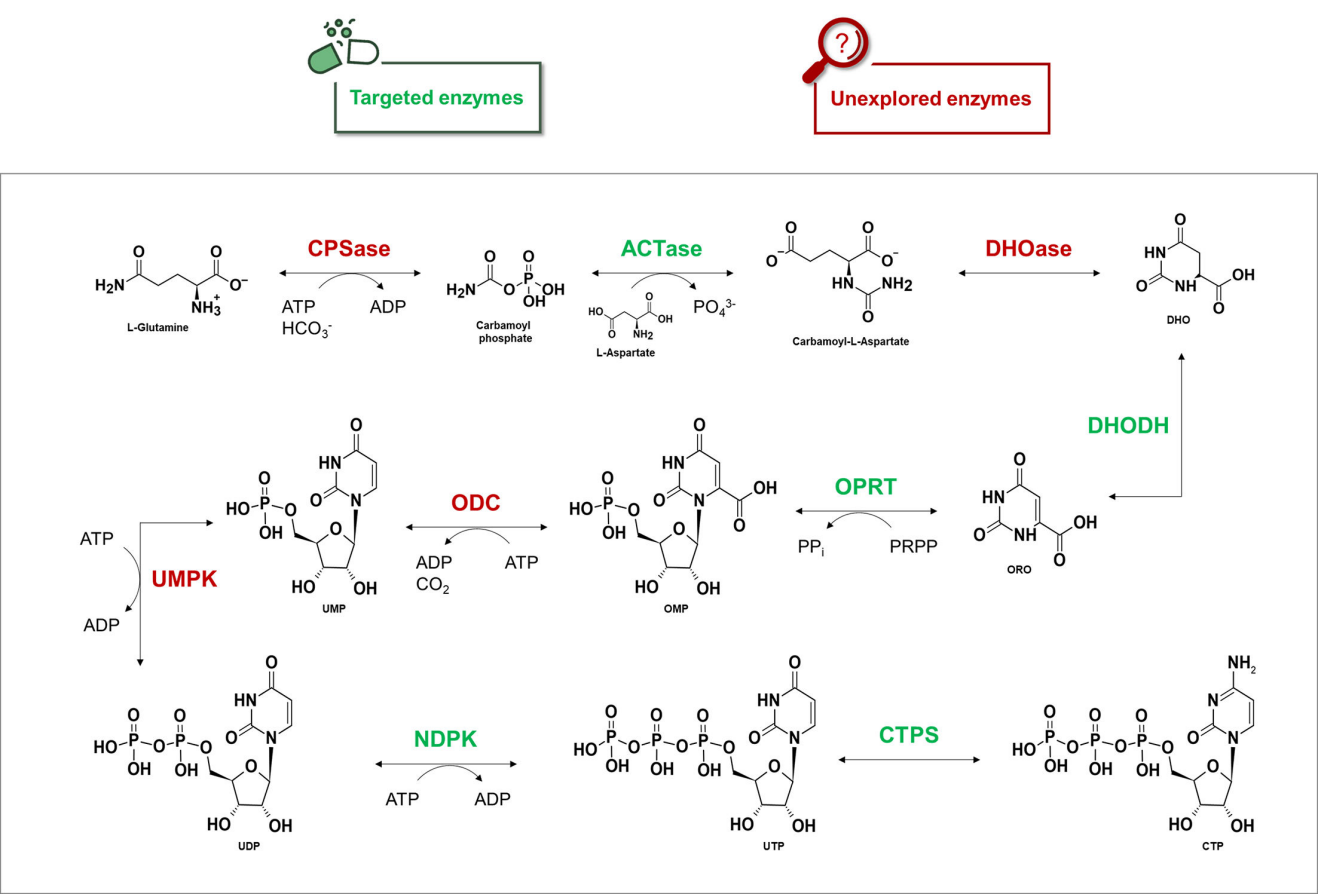
Pyrimidine *de novo* pathway in *Mycobacterium* *tuberculosis* with representation of all the enzymes involved. The green-highlighted items represent enzymes that have already been targeted in TB drug discovery, whereas the red-highlighted items indicate those that have not yet been explored in inhibition studies. This diagram illustrates the *de novo* metabolic route through which pyrimidine nucleotides are synthesized from basic precursors in MTB. Green-highlighted enzymes have already been investigated as potential drug targets in TB drug discovery. In contrast, red-highlighted enzymes represent unexplored targets, for which no inhibition studies have been reported to date. Enzymes and intermediates involved in this pathway are labeled accordingly. Abbreviations: ACT*ase*, aspartate carbamoyl transferase; ADP, adenosine diphosphate; ATP, adenosine triphosphate; CPS*ase*, carbamoyl phosphate synthase; CTP, cytidine triphosphate; CTPS, cytidine triphosphate synthetase; DHO, (S)dihydroorotate; DHODH, dihydroorotate dehydrogenase; DHO*ase*, dihydroorotase; NDPK, nucleoside diphosphate kinase; ODC, orotidine monophosphate decarboxylase; OMP, orotidine 5’-monophosphate; OPRT, orotate phosphoribosyl transferase; ORO, orotate; PPi, pyrophosphate; PRPP, 5-phospho-alpha-D-ribose 1-diphosphate; UMP, uridine monophosphate; UMPK, uridine monophosphate kinase; UDP, uridine diphosphate; UTP, uridine triphosphate.

The *de novo* PBP is a fundamental metabolic cascade with highly conserved steps across organisms. As illustrated in Figure 6, pyrimidine precursors are synthesized from l-glutamine and l-aspartate, ultimately leading to the production of UMP, a common intermediate for all pyrimidine derivatives. Table 3 lists genes encoding proteins involved in this pathway, each reported as essential for mycobacterial growth and replication *in vitro*, but only under rich medium conditions [14,80–82]. This raises important questions regarding the metabolic requirements of MTB across its life cycle. While the pathogen exhibits low metabolic demand during latent dormancy, active disease might necessitate increased metabolic activity. Therefore, the essentiality of genes encoding *de novo* enzymes observed in rich media likely reflects the enhanced proliferation, which may be attributed to the elevated metabolic needs of MTB during its active phase. Below, we describe essential enzymes involved in the *de novo* PBP of MTB (Table 3).

**Table 3:** List of gene names, corresponding protein abbreviations, MTB-reported loci, E.C. codes, UniProt codes, and the relevance of each gene for MTB survival. The final column indicates whether inhibition studies have been conducted (YES) or not (NO) for each respective enzyme. *Data derived from *Mycobrowser* database

Gene name	Protein encoded	MTB locus	E.C. code	UniProt code	Relevance*	Druggability
*carA carB*	CPS*ase*	Rv1383 Rv1384	6.3.5.5 6.3.4.16	P9WPK5 P9WPK3	Essential gene [14,80–82]	NO
*pyrB*	ACT*ase*	Rv1380	2.1.3.2	P9WIT7	Essential gene [14,80–82]	YES [83–85]
*pyrC*	DHO*ase*	Rv1381	3.5.2.3	P9WHL3	Essential gene [14,80–82]	NO
*pyrD*	DHODH	Rv2139	1.3.5.2	P9WHL1	Essential gene [14,80–82]	YES [86–88]
*pyrE*	OPRT	Rv0382c	2.4.2.10	P9WHK9	Essential gene [14,80–82]	YES [89,90]
*pyrF*	ODC	Rv1385	4.1.1.23	P9WIU3	Essential gene [14,80–82]	NO
*pyrH*	UMPK	Rv2883c	2.7.4.22	P9WHK5	Essential gene [14,80–82]	NO
*ndkA*	NDPK	Rv2445c	2.7.4.6	P9WJH7	Essential gene [14,80–82]	YES [50–58]
*pyrG*	CTPS	Rv1699	6.3.4.2	P9WHK7	Essential gene [14,80–82]	YES [91–94]

#### Carbamoyl phosphate synthase-aspartate carbamoyl transferase (ACT*ase*)-Dihydroorotase: the CAD complex

Each enzymatic step constituting the *de novo* PBP is conserved across eukaryotes and prokaryotes. However, where prokaryotes engage monofunctional enzymes, eukaryotes usually mount complex macromolecular machinery based on chimeric proteins in which different domains are connected by polypeptide linkers. This applies to the human multifunctional carbamoyl-phosphate synthetase 2, Aspartate transcarbamoylase, and Dihydroorotase enzyme (CAD), encoded solely by the *cad* gene [95,96]. In MTB, CPS*ase*, ACT*ase,* and DHO*ase* are encoded by the *carA/B*, *pyrB,* and *pyrC* genes, with these three domains functioning independently in the synthesis of DHO. The rate-limiting step of the entire cascade is represented by the conversion of DHO into ORO, catalyzed by the DHODH enzyme. Most of the drug discovery studies have been performed on DHODH and downward enzymes, while CAD reactivity has not been considered a therapeutic opportunity, except for ACT*ase*, described below.

##### Aspartate carbamoyl transferase

ACT*ase* catalyzes the condensation of carbamoyl phosphate and l-aspartate to produce n-carbamoyl-l-aspartate and inorganic phosphate. In MTB, ACT*ase* corresponds to the catalytic C-terminal domain of the larger eukaryotic CAD complex. The ACT*ase* of *Mycobacterium smegmatis*, often used as a reference in TB drug discovery, is inhibited by ATP, CTP, and UMP nucleotides. Additionally, succinate and maleate, dicarboxylic acid analogs of l-aspartate, function as competitive inhibitors of the enzyme’s activity [83]. Sequence alignment between MTB and human ACT*ase* reveals a 32% sequence identity, an important factor to consider in drug design. Conserved sites among different species are typically associated with substrate/cofactor binding, presenting a challenge for selective drug development. In this scenario, Du et al. [84] developed a series of compounds targeting an allosteric pocket, achieving species selectivity among *Plasmodium falciparum*, human*,* and MTB ACT*ase,* with IC_50_ values in a single-digit μ M range. Moreover, the most potent inhibitors demonstrated a minimum inhibitory concentration (MIC) against the H37Rv strain in the low μ M range. These results underscore the potential of MTB ACT*ase* as an innovative target, demonstrating the feasibility of species-selective inhibition. However, structural data for this enzyme are still missing and, given its critical role in regulating *in vivo* nucleotide concentrations, future drug discovery efforts can leverage these findings as a foundation for rational and selective design strategies targeted at MTB ACT*ase* [85].

### Dihydroorotate dehydrogenase

**Figure 7:**
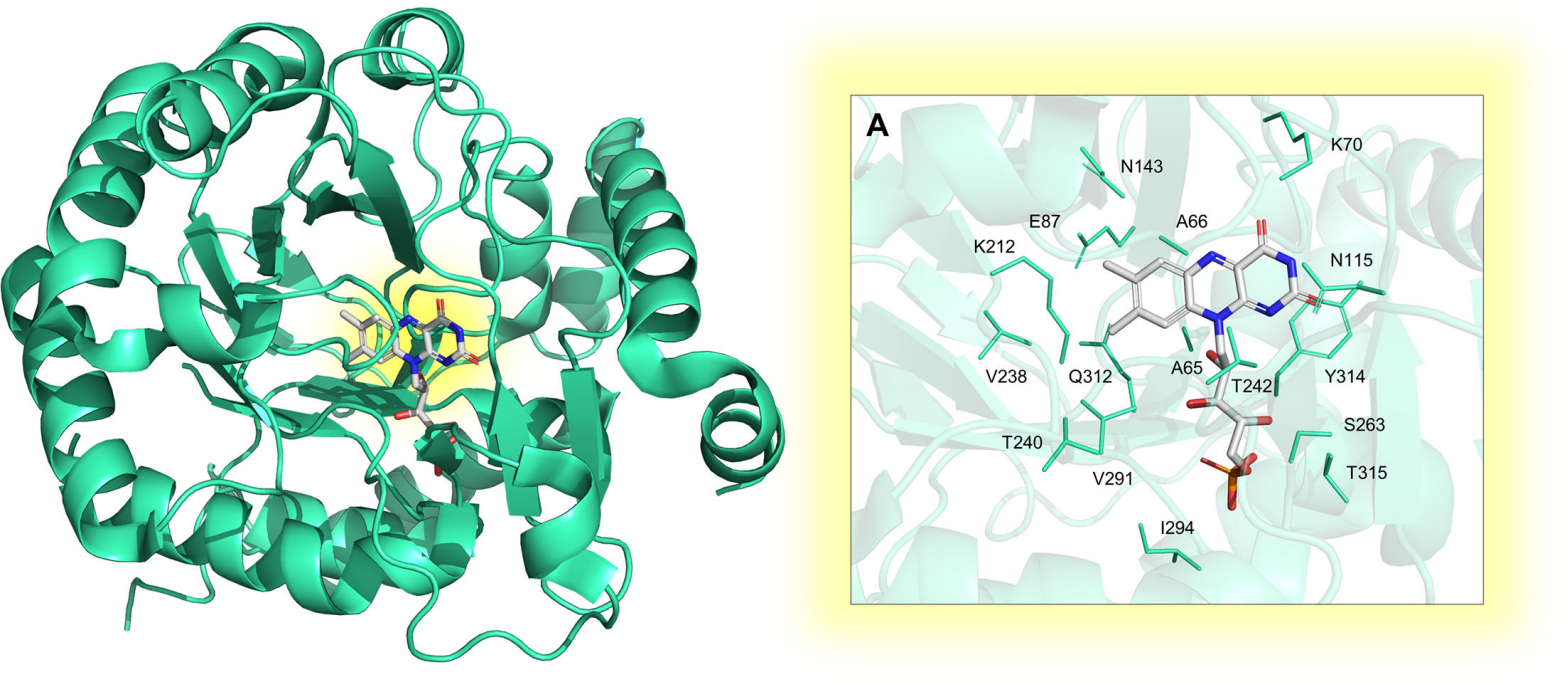
Experimental structure of the monomeric MTB DHODH enzyme. The structure was solved by X-ray crystallography in complex with: (**A**) the cofactor FMN (PDB: 8OFW [88] located in the catalytic tunnel (highlighted as a yellow spot in the structure).

DHODH oxidizes DHO to ORO in a ‘ping-pong’ reaction [97] involving flavin mononucleotide (FMN) and menaquinone (MQ) [98] as cofactors. According to protein sequence, substrate preferences, and metabolic cell state, DHODHs are classified into cytosolic class I DHODHs (gram-positive bacteria and lower eukaryotes) and membrane-bound class II DHODHs (Gram-negative bacteria and higher organisms, including human and MTB DHODH [99]. In class II DHODHs, catalysis proceeds following two half-reactions: the catalytic serine deprotonates C(5)-DHO, transferring a hydride to FMN [100]; reduced FMNH_2_ is re-oxidized by a quinone molecule through hydride transfer. In human DHODH, quinol is ultimately recruited from the mitochondria to feed the electron transport chain. Inhibition studies led to the discovery of effective human DHODH inhibitors for the treatment of several diseases [86,101–112]. Despite its essential role in bacilli viability, MTB DHODH has not been extensively characterized in terms of inhibition studies. Teixeira et al. [87] first characterized the enzyme using a partial crystal model and identified Q_0_ as a non-competitive inhibitor (K_i_ 138 μ M) [88]. Given the strict conservation of residues in the DHO/ORO pocket across diverse species, the presence of non-conserved residues in the quinone pocket offers unique opportunities for ensuring selective drug design. Our previous work reported the first crystallographic structure of the full-length MTB DHODH [89] in complex with the cofactor FMN (Figure 7 and A), coupled with a biochemical investigation of the protein [K_M_(DHO) 21.1 μ M; K_M_(MQ) 54 μ M]. We also identified, through a structure-based inhibitor screening, a selective quinone-scaffold inhibitor with fluorescent properties, potentially instrumental to *in vitro/vivo* imaging studies [89]. Despite its fundamental role, MTB DHODH remains poorly characterized, presenting an opportunity for the scientific community to address this gap and facilitate the discovery of innovative antitubercular candidates.

### Orotate phosphoribosyl transferase (OPRT)

**Figure 8:**
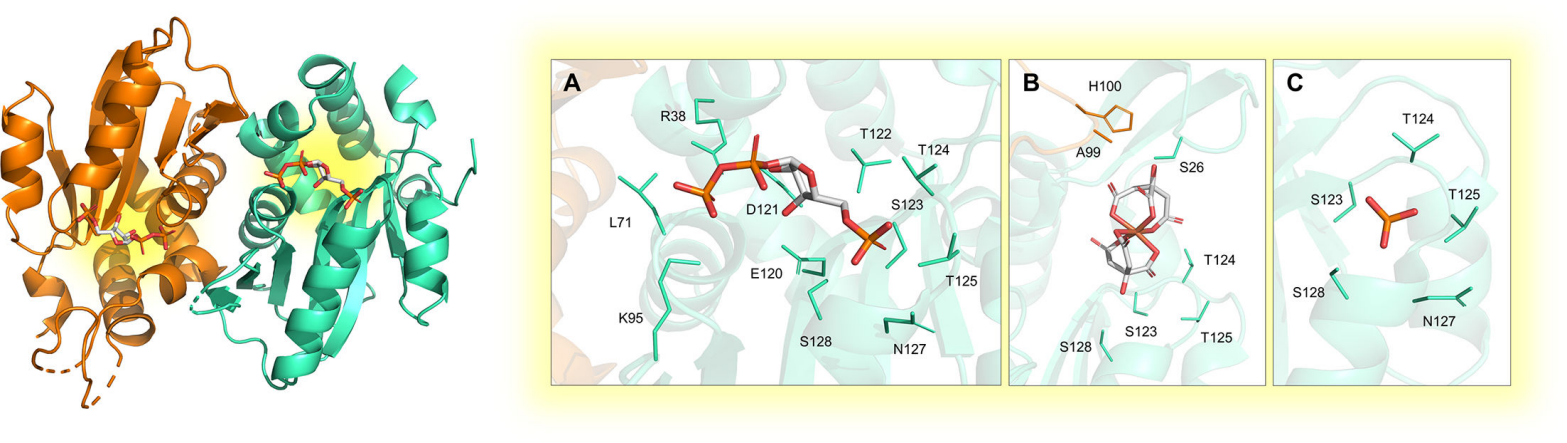
Experimental structure of the homodimeric MTB OPRT enzyme. The structures were solved by X-ray crystallography in complex with: (**A**) PRPP (PDB: 5HKF [114]); (**B**) Fe(III)-dicitrate (PDB: 5HKI [114]); (**C**) inorganic phosphate (PBD: 5HKL [114]) in the cofactor binding site (highlighted as a yellow spot in the structure).

OPRT catalyzes the transfer of a ribosyl phosphate group from PRPP to ORO, forming OMP. The Mg^2+^-dependent reaction follows a mono-iso ordered Bi-Bi kinetic mechanism [113,114] and represents the final step in *de novo* PBP, with no contribution from pyrimidine *salvage*, making it essential for MTB survival. Our group has elucidated the original crystal structure of the functional homodimeric protein through X-ray diffraction in complex with PRPP (Figure 8A), Fe(III)dicitrate (Figure 8B), and inorganic phosphate (Figure 8C), providing a foundation for rational and selective drug design [90]. Mechanistic investigation of OPRT catalysis gave valuable insights into its mode of action, facilitating the development of loss-of-function or gain-of-function molecules to explore its biological role [115]. Sequence alignment indicates a 31% identity between human and MTB OPRT, with an RMSD of 1.373 Å for structural superposition. Although most active site residues are conserved, differences such as the V125/T121 substitution and the flexible R22-E30 loop define species-specific features relevant for ligand recognition and selective drug design. Previous studies targeted this enzyme for malaria [116,117] and toxoplasmosis [91] treatments, and given its role in MTB survival, it represents a promising target for the development of novel antitubercular therapies.

### Cytidine triphosphate synthetase (CTPS)

**Figure 9:**
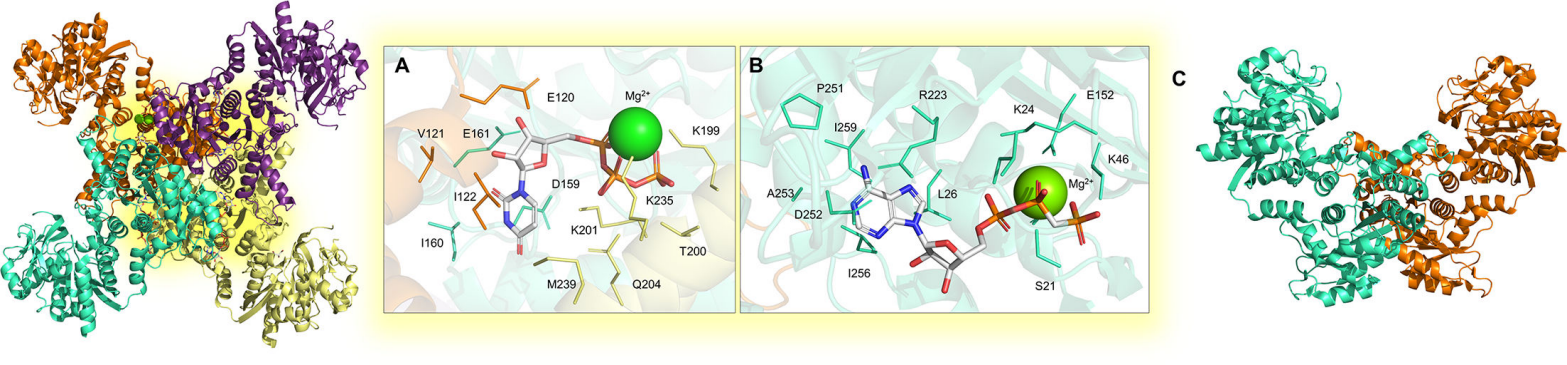
Experimental structure of the MTB CTPS enzyme. The structures were solved by X-ray crystallography with the enzyme in its: (**A**) homotetrameric form in complex with Mg^2+^ and UTP (PDB: 4ZDJ [91]; (**B**) homotetrameric form in complex with Mg^2+^, UTP, and phosphomethylphosphonic acid adenylate ester (PDB: 4ZDK [91]); (**C**) homodimeric apo form (PDB: 4ZDI [91]).

CTPS catalyzes the ATP-dependent amination of UTP to obtain CTP, using either ammonia or l-glutamine as nitrogen sources. Inhibition of CTPS decreases CTP levels, affecting DNA/RNA biosynthesis and multiple metabolic processes, including lipids, carbohydrates, and amino acids biosynthesis, as well as cAMP-dependent signaling [92]. Down-regulation of *pyrG* significantly impairs MTB growth, highlighting its crucial role in MTB survival [93]. The enzyme regulates the intracellular concentration of CTP by finely distinguishing between uracil or cytosine moieties, with GTP acting as an activator when glutamine serves as the nitrogen donor, stabilizing the tetrahedral intermediates, while CTP works as an allosteric inhibitor. Given its essential role in both catabolic and anabolic processes in MTB, the druggability of CTPS has been extensively analyzed for antitubercular drug discovery. High-throughput screening identified thiophenecarboxamide derivatives 7904688 and 7947882 with potent activity (MICs around 0.5 μ g/ml) against replicating, non-replicating, and intracellular MTB [94]. Both molecules, activated by EthA monooxygenase and show no cytotoxicity below 40 μ g/ml against human cell lines. Resistant mutants cultivated at suboptimal doses of these compounds (10 μ g/ml) displayed mutations in *ethA* and *pyrG*, confirming their role as targets. The experimentally determined crystal structure revealed a homotetrameric assembly driven by nucleotide interactions that promote tetramerization [92] (Figure 9A and B). Supporting this, in the absence of nucleotides, the protein is organized as a homodimer [92] (Figure 9C), with each homodimer representing half of the functional tetramer. In 2017, following the same phenotypic screening approach, three potent compounds endowed with a 4-(pyridin-2-yl)thiazole group were identified from a library of 117 molecules. These inhibitors, namely SK1570606A, GSK920684A, and GSK735826A, demonstrated MICs, IC_50_, and K_i_ values all in the low μ M range, indicating competitive inhibition toward the ATP-binding site[118]. Despite these compounds also inhibiting human CTPS, the absence of toxicity towards human cell lines provides a promising foundation for the development of selective MTB inhibitors that do not affect host homeostasis.

## Conclusions

**Figure 10:**
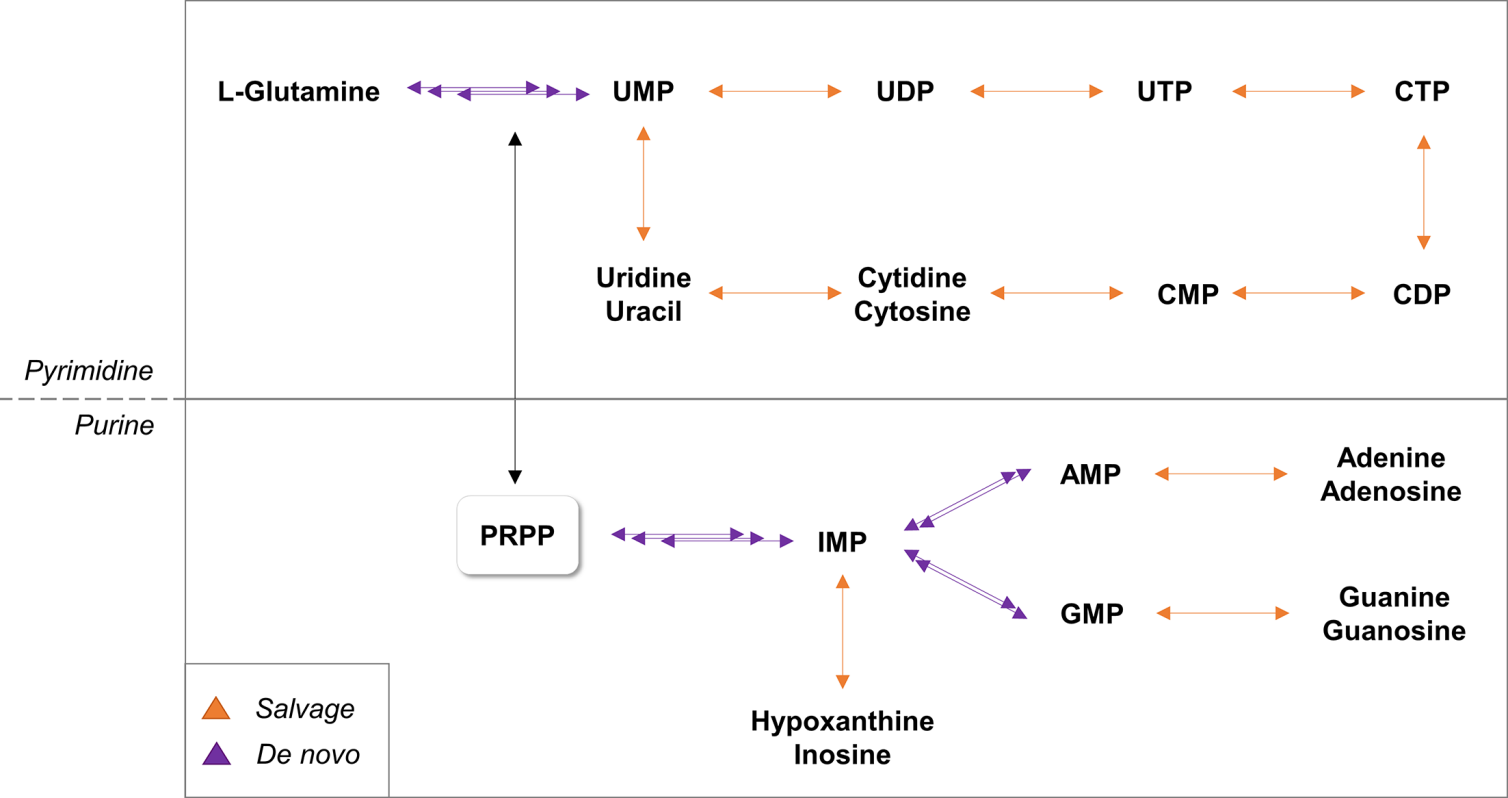
Schematic representation of the pyrimidine and purine biosynthesis pathways. Orange arrows depict reactions belonging to the *salvage* routes, whereas violet arrows indicate those of the *de novo* pathways. The diagram highlights the central role of PRPP as a shared intermediate and key regulatory node connecting the two metabolic routes. Abbreviations: AMP, adenosine monophosphate; CMP, cytidine monophosphate; CDP, cytidine diphosphate; CTP, cytidine triphosphate; GMP, guanosine monophosphate; IMP, inosine monophosphate; PRPP, 5-phospho-alpha-D-ribose 1-diphosphate; UMP, uridine monophosphate; UDP, uridine diphosphate; UTP, uridine triphosphate.

MTB infection remains a global health emergency, exacerbated by the rise of drug-resistant strains. This underscores the urgent need for new therapeutic agents. MTB has the complete repertoire to either synthesize nucleotides *de novo* or scavenge them from the host, providing the essential building blocks for DNA/RNA. Since anabolic pyrimidine pathways are generally essential for bacterial survival and involve enzymes with low homology to human counterparts, they represent a promising and selective source of drug targets. The two enzymatic cascades described in this work (i.e. *de novo* and *salvage*) are activated at different stages of the bacterial cell cycle, often self-compensating. This opens the potential for synthetic lethality as a therapeutic strategy. In MTB, purine and pyrimidine metabolism are closely interconnected, and their cross-talk reshapes across native, latent, and active disease states. Under standard nutrient-rich conditions, both pathways rely heavily on the shared precursor PRPP, which feeds the *de novo* as well as *salvage* reactions, thereby creating an intrinsic metabolic link between the two (Figure 10) [119,120]. For instance, the PRPP-synthetase, namely MTB PrsA (encoded by the *prsA* gene), has been extensively validated as a robust target for the development of antibacterial agents [121,122].

It is still not clearly defined how the *de novo* or *salvage* processes are regulated during latent or active infections, and the available data remain quite limited. Nevertheless, some studies hypothesize that during latent infection, characterized by hypoxia and nutrient deprivation, the *de novo* synthesis may be attenuated, possibly due to restricted energy and precursor availability, while the *salvage* pathway might become more relevant for maintaining minimal nucleotide pools [123–126]. In contrast, during active disease, MTB appears to re-engage more energy-demanding pathways to sustain the balanced production of nucleotides needed for efficient DNA replication [89,127,128]. Overall, the dynamic cross-talk between these pathways ensures that MTB can flexibly shift between replication and persistence, offering multiple opportunities for therapeutic intervention.

A recent study [129] explored an innovative antitubercular strategy by inhibiting PurF, a key enzyme involved in the *de novo* purine biosynthesis pathway, demonstrating a critical metabolic vulnerability in the *de novo* and *salvage* biosynthesis. Particularly noteworthy was the authors’ phenotypic screening approach: following administration of suboptimal doses of the candidate compound JNJ-6640 (MIC_90_ against MTB: 8.6 nM), resistant bacterial strains were selected. Whole-genome sequencing of these resistant clones identified four distinct single-nucleotide polymorphisms (I241V, F428C, F428V, and S470F) within the *purF* gene, all conferring resistance to the compound. This work not only validates PurF as a druggable target but also highlights the inability of the *salvage* pathway to compensate for the *de novo* biosynthesis. This conceptual framework could be extended from the purine to the pyrimidine pathway, thereby supporting the therapeutic potential of targeting *de novo* PBP enzymes in TB treatment.

Host-directed therapies (HDTs) are an emerging alternative, aiming to modulate the host’s immune and inflammatory responses rather than directly target the pathogen. While promising, HDTs require careful target selection to avoid immunosuppressive effects, especially in TB patients co-infected with HIV-1. Despite encouraging preclinical results, robust clinical data remain limited [130–133].

To streamline our literature review, we analyzed the state-of-the-art in research on the inhibition of enzymes directly involved in the PBP of MTB, with a particular emphasis on essential proteins for which there have been rational design studies concerning molecules of pharmaceutical interest. This understanding may ultimately support innovative drug discovery efforts aimed at treating human TB.

**Perspectives**

- **Bulleted 1 - Highlight the importance of the field -** discovery of new therapeutic targets for treating tuberculosis (TB) is a critical public health priority, given the high mortality rate associated with *Mycobacterium tuberculosis* (MTB) infections. Consequently, there is an urgent need for the development of novel therapies that not only effectively fight resistant strains but also exhibit minimal toxicity to host human cells.
- **Bulleted 2 - Summary of the current thinking** ***-*** current treatment regimens are hindered by low patient adherence, and the always increasing number of drug-resistant MTB strains further complicates efforts to control and eradicate the disease. Targeting enzymes involved in metabolic pathways essential for MTB, with minimal impact on the human host, needs to be explored.
- **Bulleted 3 - Comment on future directions** ***-*** new molecular entities are being investigated with the aim of selectively binding and inhibiting the pathogen's enzymes with low toxicity to human cell lines. Structural, biochemical, and microbiological investigations will continue with a multidisciplinary and integrated approach to find the optimal inhibitor for anti-TB drug discovery.

## Open Access

This article has been published open access under our Subscribe to Open programme, made possible through the support of our subscribing institutions, learn more here: https://portlandpress.com/pages/open_access_options_and_prices#conditional

## Abbreviations

ACTase: aspartate carbamoyl transferase
ADP: adenosine diphosphate
AMP: adenosine monophosphate
ATP: adenosine triphosphate
CAD: Carbamoyl-phosphate synthetase 2, Aspartate transcarbamoylase, and Dihydroorotase
CDA: cytidine deaminase
CDP: cytidine diphosphate
C2H4folate: 5,10-methylenetetrahydrofolate
CMP: cytidine monophosphate
CMPK: cytidine monophosphate kinase
COVID-19: Coronavirus Disease 19
CPSase: carbamoyl phosphate synthase
CTP: cytidine triphosphate
CTPS: cytidine triphosphate synthetase
DHO: (S)dihydroorotate
DHODH: dihydroorotate dehydrogenase
DHOase: dihydroorotase
FAD: flavin adenine dinucleotide
FADH2: reduced flavin adenine dinucleotide
FMN: flavin mononucleotide
GMP: guanosine monophosphate
HDTs: host-directed therapies
H2folate: dihydrofolate
IMP: inosine monophosphate
MIC: minimum inhibitory concentration
MQ: menaquinone
MTB: *Mycobacterium tuberculosis*
NADP+: nicotinamide adenine dinucleotide phosphate
NADPH: reduced nicotinamide adenine dinucleotide phosphate
NDPK: nucleoside diphosphate kinase
ODC: orotidine monophosphate decarboxylase
OMP: orotidine 5’-monophosphate
OPRT: orotate phosphoribosyl transferase
ORO: orotate
PBP: pyrimidine biosynthetic pathway
PDB: Protein Data Bank
PPi: inorganic pyrophosphate
PRPP: 5-phospho-alpha-D-ribose 1-diphosphate
PyNP: pyrimidine nucleoside phosphorylase
SAR: structure-activity-relationship
TB: tuberculosis
TSA: thymidylate synthase thyA
TSX: thymidylate synthase thyX
UDP: uridine diphosphate
UMP: uridine monophosphate
UMPK: uridine monophosphate kinase
UPRTase: uracil phosphoribosyltransferase
UTP: uridine triphosphate
dCDP: deoxycytidine diphosphate
dCMP: deoxycytidine monophosphate
dCTP: deoxycytidine triphosphate
dCTPase: deoxycytidine triphosphate deaminase
(d)NDPs: nucleoside diphosphates
(d)NTPs: nucleoside triphosphates
dTDP: deoxythymidine diphosphate
dTMP: deoxythymidine monophosphate
dTMPK: deoxythymidine monophosphate kinase
dTTP: deoxythymidine triphosphate
dUDP: deoxyuridine diphosphate
dUMP: deoxyuridine monophosphate
dUTP: deoxyuridine monophosphate
dUTPase: deoxyuridine triphosphatase
